# The Microbiome-Immune Axis Therapeutic Effects in Cancer Treatments

**DOI:** 10.4014/jmb.2208.08002

**Published:** 2022-08-19

**Authors:** Young Min Son, Jihwan Kim

**Affiliations:** Department of Systems Biotechnology, Chung-Ang University, Anseong 17546, Republic of Korea

**Keywords:** Microbiome, immune responses, cancers, immunotherapy, tissue microorganisms, microbiome-immune axis

## Abstract

During the last decades, research and therapeutic methods in cancer treatment have been evolving. As the results, nowadays, cancer patients are receiving several types of treatments, ranging from chemotherapy and radiation therapy to surgery and immunotherapy. In fact, most cancer patients take a combination of current anti-cancer therapies to improve the efficacy of treatment. However, current strategies still cause some side effects to patients, such as pain and depression. Therefore, there is the need to discover better ways to eradicate cancer whilst minimizing side effects. Recently, immunotherapy, particularly immune checkpoint blockade, is rising as an effective anti-cancer treatment. Unlike chemotherapy or radiation therapy, immunotherapy has few side effects and a higher tumor cell removal efficacy depend on cellular immunological mechanisms. Moreover, recent studies suggest that tissue immune responses are regulated by their microbiome composition. Each tissue has their specific microenvironment, which makes their microbiome composition different, particularly in the context of different types of cancer, such as breast, colorectal, kidney, lung, and skin. Herein, we review the current understanding of the relationship of immune responses and tissue microbiome in cancer in both animal and human studies. Moreover, we discuss the cancermicrobiome-immune axis in the context of cancer development and treatment. Finally, we speculate on strategies to control tissue microbiome alterations that may synergistically affect the immune system and impact cancer treatment outcomes.

## Introduction

Cancer, one of the foremost reasons of deaths throughout the world, is a disease whereby some of body's cells grow uncontrollably and spread to other parts of the body. In 2020, more than 1.8 million new cancer cases were estimated to be diagnosed and around 606,520 of people died in the United States alone [1]. An enormous variety of cancer types have been reported and studied worldwide. The most common in humans are breast, lung, colon, rectum, and prostate cancers. There are several causes for cancer development in humans, including tobacco use, high body mass index, alcohol consumption, unbalance food intake and lack of physical activity [2, 3]. Moreover, there are also biological carcinogens, often carried by microorganisms such as viruses, bacteria, or parasites, reported as the major reasons of cancer development [4]. Despite the massive efforts made in the cancer research field and the remarkable progress seen at the clinical level, cancer treatment and prevention are yet intangible. Besides its urgent need for treatment, cancer dynamics are highly related to the understanding of life itself from genetic to ecological perspectives and a better understanding of cancer per se is crucial for greater knowledge of human biology and scientific advancement. However, due to their cardinal feature of evading the immune system, which delay the design of effective anticancer therapeutic strategies, further advancement is needed for current cancer therapies.

Regarding cancer persistence, numerous studies have reported that several factors contribute to the tumor persistence in a steady state immune system. The equilibrium and senescence of patients’ immune systems are considered two key mechanisms underlying cancer detection during immunological surveillance [5]. Another possible mechanism influencing cancer immune avoidance is likely related to regulatory T (Treg) cell function or their anti-inflammatory cytokine secretions. These cells are responsible for modifying the production of immune suppressive mediators, tolerance and immune deviation [6-9]. Therefore, it is necessary the development of strategies that enhance immune responses under cancer environments to promote the efficacy of current cancer therapies.

Recently, microbiota indicating varied microorganisms such as bacteria, virus, fungi and archaea in most living organisms has been shown to exert great potential in affecting human health and disease, particularly controlling the immune system [10-12]. Over the last few decades, the microbiome research field has been rapidly evolving and has become a topic of great scientific and public interest, with the development of remarkable tools, such as next-generation sequencing (NGS) techniques, for genomic studies. In the past several years, scientific research on the relationship of the microbiome and host immunity has grown exponentially. Strikingly, studies have found that, on one hand, the microbiome crucially shapes the development of innate and adaptive immunity and, on the other hand, the immune system itself influences the microbiome [13-15].

Despite the existence of many studies in the past decades demonstrating the relationship of cancer therapies and the immune system, our knowledge on the use of the host microbiome to improve immune responses under cancer environment remains limited, particularly regarding solid tumors. Furthermore, little is known about the changes of microbiome under cancer environments. Herein, we highlight recent evidence describing the roles of the microbiome and the immune system in cancer pathophysiology, reviewing knowledge that can be applied to improve cancer therapies.

## Microbiome: General Facts and Its Role in Cancer

### Microbiome in Different Tissues

Microbiota encompasses a community of microorganisms, including commensal, symbiotic or pathogenic, living in a specific environment or organism [16, 17]. In healthy humans, microbiota such as bacteria, viruses and fungi have been found in most organs including the mouth, throat, nasal cavity, gut, lung, and vagina [18, 19]. Recently, advancements in genetic technology have supported the development of microbiome studies focused on the communication and genome information of microbiota in specific microenvironments [20-22]. In fact, during the last decade, the development of NGS techniques, including 16S rRNA gene sequencing, RNA sequencing, and shotgun metagenomic sequencing, remarkably enhanced our knowledge about the microbiome, allowing the characterization of unculturable microorganisms [21, 23].

The human intestine harbors the most abundant microbiome, composed of a great number and wide variety of species and metabolites compared to other organs [24-26]. The most striking feature of the gut microbiome is that gut microbiome can affect the maintenance and/or function of other tissues [27-29]. For example, serotonin, a powerful neurotransmitter involved in mood regulation, can be synthesized in the gut microbiota and transmitted into the brain through the “gut-brain axis” [30].

Regarding the lung microbiome, the *Proteobacteria* is one of the most dominant phyla and lung has distinct features compared to other microbial groups in the oral and nasal cavities, gut, skin, and vagina. It has been previously reported that the alpha diversity of lung microbiota, a measure of microbiome diversity within a local scale, can be influenced by environmental factors such as air particles, the density of residency populations, history of tobacco smoking or of chronic bronchitis [31].

*Lactobacillus* including *L. crispatus*, *L. gasseri*, *L. iners* and *L. jensenii* is common species in vaginal area. Lactic acid, the major product from *Lactobacillus*, maintains lower pH in the vaginal area, and together with bacteriocins and hydrogen peroxide it provides the protective function against foreign pathogens [32, 33]. Moreover, *lactobacillus* have been reported that it can induce the differentiation of regulatory T cells (Tregs) to prevent excessive inflammation in this area [34].

Overall, each human organ has its own set of microbial species because they provide different ideal environments, with specific factors such as high oxygen, insufficient nutrients, and acidic surface, for microbiota to live in. Therefore, in the next subsections we will further investigate the roles of tissue-specific microbiome in different cancer environments.

### Microbiome in Different Types of Cancers

**Fig. 1:**
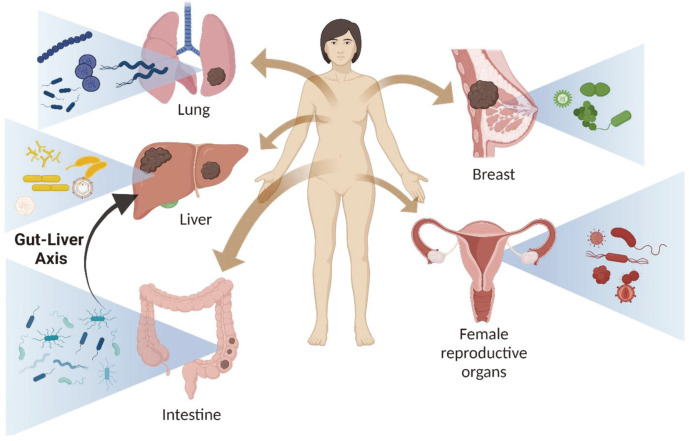
Varied microbiota composition in several human organs under the cancer environment. HPV is dominant microbe in the breast cancers and *Proteobacteria*, *Firmicutes*, and *Actinobacteria* were also reported as the major phyla in the patient who have a breast cancer. Similar to breast cancer, HPV has been found in vaginal area as well and it has been reported that it might cause the vaginal carcinogenesis. Due to the functional feature of lung which consistently exchange the air, lung has a distinct microorganism content like a significant increase of *Granulicatella*, *Abiotrophia*, and *Streptococcus* genera. Intestine area includes most diversity of microbiota than other organs and it can affect other tissue’s microbiota composition. Certain metabolites from microbiota of intestine transfer into liver then might regulate inflammation and hepatotoxicity in the liver via Gut-liver axis.

Several recent studies have indicated a critical role of the microbiome in different tissues for numerous diseases, including the formation and development of cancer (Fig. 1) [35-37]. As mentioned in the previous section, current “omics” technologies, such as transcriptomics, proteomics, metabolomics, and metagenomics, have enabled a richer and deeper understanding of the relationship between the microbiome and specific-tissue cancer development and progression.

The report of global cancer statistics shows that colorectal cancer was ranked a third in new incidences and second in deaths [38]. A large number of microbiota is observed in the intestinal area and it shows a close interaction with this organ to contribute to energy harvesting, adjusting immune system and metabolisms [39-42]. Early animal studies of colorectal cancer demonstrated that specific microbiota including species of *Escherichia*, *Enterococcus* and *Bacteroides* were involved with developing colorectal carcinogenesis in germ-free and conventional mice models [43-45]. Gut dysbiosis is one of the critical reasons to promote cancer development via induction of chronic inflammation by pathogenic microbiota infiltration into organ. Enterotoxigenic *Bacteroides fragilis* (ETBF) is involved with the tumor formation by producing toxin which induces a chronic inflammation [46]. *Escherichia coli* (*E. coli*) promotes the gut permeability, resulting increase the pathogenic bacteria infiltration into mucosa area [47]. These results suggest that microbiota in cancer environment is closely related to gut disease progress.

The lung is one of the human organs that is most consistently exposed to external microbial stimuli. Evidence shows that the lung has a distinct microbiome composition, a key factor that may contribute to the development of lung cancer [48, 49]. When compared to healthy control samples, tissue samples from lung cancer patients were found to have a significant increase of *Granulicatella*, *Abiotrophia*, and *Streptococcus* genera, despite the apparent decreased diversity of microbiota [31, 50-52]. One study has reported that specific bacterial taxon may contribute to the development of lung cancer, with a high relative abundance of *Thermus* genus being observed in tissue samples from subjects with advanced tumor stages, and a high relative abundance of *Legionella* genus being dominant in patients who developed metastases [31]. In saliva samples from lung cancer patients, *Veillonella* and *Capnocytophaga* genera, which are usually present in oral sites, were shown to be dramatically higher, being considered early detective biomarkers of small cell carcinoma and adenocarcinoma [53]. Furthermore, a relative abundance of *Veillonella* and *Megasphaera* genera has been reported in bronchoalveolar lavage samples of lung cancer patients compared to healthy control [54]. *Granulicatella adiacens* and other opportunistic pathogens were also found in sputum samples of lung cancer patients [52]. Overall, it is hard to define specific bacterial taxa associated with lung cancer due to the variability in the type of patients and in the sample collection methods. However, certain species of microbiota which persisting with consistent characters; the enhancement of total abundance of microbiota, decreased alpha diversity, and altered bacterial composition have been observed in the lung cancer patients. Although the effects of an altered bacterial diversity in lung cancer patients have not been elucidated yet, a recent article showed that an enhanced alpha diversity has a positive correlation with the survival ratio of patients as well as with better effects of treatments on both cervical cancer and resected pancreatic adenocarcinoma [55, 56].

The dangerous relationship between HPV infections and the vaginal microbiome has been reported to contribute to vaginal carcinogenesis [57-60]. Recent evidence, obtained through 16S rRNA sequencing, shows that certain anaerobic microbes are abundant within the vaginal microbiome of HPV-infected patients, suggesting that specific changes in microbiome composition may be utilized as a biomarker to assess the presence of HPV and identify alterations in the cervical microenvironment [57]. Similarly, microbes belonging to *Prevotella*, *Porphyromonas*, and *Enterococcus* genera were found to be increased in the HPV-infected cervical environment, whereas the relative abundance of *Bacteroides* genus was decreased [61]. Gene expression studies involving patients with cervical lesions are crucial to identify the critical relationship between HPV infections and cervical cancer. For instance, a recent report shows that the expression of toll-like receptor 4 (TLR4) is closely related to HPV infection and vaginal cancer cell growth, with TLR4 signaling contributing to the formation of a local immunosuppressive microenvironment in the vaginal area [60]. Another study demonstrates that both the human immunodeficiency virus (HIV) and the herpes simplex virus (HSV) are associated with the formation of cervical cancers, suggesting that the increase of microbiota diversity and cervicovaginal inflammation in HIV or HSV seropositive patients may adversely impact genital health [58].

Various recent studies have revealed the composition of the microbiome in breast cancer tissue. Specifically, the *human papillomavirus* (HPV) is one of the most common microorganisms present in breast cancers when compared to other normal breast controls [60, 62, 63]. Interestingly, several studies suggest that the HPV might be a critical trigger for breast ductal carcinomas due to its capacity to immortalize resident epithelial cells [64-66]. Besides HPV, live bacteria from three main phyla, including *Proteobacteria*, *Firmicutes*, and *Actinobacteria*, are found in breast cancer tissues [67]. Additionally, results obtained from the correlation analysis of expression profiles in samples from breast cancer patients show a strong association between *Haemophilus influenzae* and proliferation pathways genes, namely G2M checkpoint and E2F transcription factors [68]. These data suggests that alterations in bacterial diversity may influence the host immune response and, therefore, lead to positive outcomes in cancer patients.

## Microbiome and Immune Axis

Several reports have demonstrated the clear relationship between the microbiome and the immune system, including both innate and adaptive immune responses. Early studies using germ-free (GF) animals provided evidence suggesting the connection between microbial exposure and the development of an immune system [13, 69]. Indeed, GF mice were found to exhibit increased vulnerability to infections. However, when microbiota from standard pathogen free (SPF) mice was transplanted into GF mice, immunodeficiency was overhauled and returned to normal levels, with immune maturation taking place. Regarding innate immunity, gut microbiota demonstrated its ability to enhance myelopoiesis and myeloid cell maturation, induce functional innate lymphoid cells [13, 70]. All these studies suggest that microbes are engaged in the maturation of both innate and adaptive immunity. Many epidemiological studies have also supported the idea that the immune development is critically shaped by the microbiome. In the processes of carcinogenesis and cancer progression, the microbiome contributes to the alteration of the immune system to manipulate and regulate the crosstalk between the immune system and the tumor. In the following subsections, the microbial influence on each type of immunocyte during cancer development will be reviewed.

### Dendritic Cells (DCs)

DCs are located in the basement membrane of mucosal tissues and represent the first line of defense against microbes, functioning as the most efficient antigen-presenting cells (APCs) that trigger adaptive immunity. One study has shown that gut microbiota TLR4-mediated signaling induces the activation of DCs and the activation of adoptively transferred tumor-specific CD8^+^ T cells in melanoma mice models [71]. Similarly, treatment of vancomycin an antibiotic that mainly acts on gram-positive bacteria in the gut, was found to enhance the cross-presentation of tumor-associated antigen (TAA) on DCs and to promote the activation of cytolytic CD8^+^ T cells [72]. The composition of microenvironment and microbiota-derived cues are critical to program conventional DCs during steady-state conditions for proper immune response. Particularly, microbiota was found to be required for the constitutive production of type I interferons (IFN) by plasmacytoid DCs, thus triggering early immune responses against pathogen invasions [73].

### Natural Killer (NK) T Cells

NKT cells, which are engaged in anti-cancer cytotoxic immune responses [74], are also affected by the microbiome. It was found that gut bacteria that metabolize primary into secondary bile acids hamper the immunological surveillance of liver cancers through the chemokine-dependent accumulation of hepatic NKT cells. In fact, when vancomycin was applied to modulate the gut microbiome, NKT cells were activated to promote anti-tumor immune responses [75]. Conversely, another report shows that invariant NKT (iNKT) cells can shape gut microbiota during intestinal inflammation. Indeed, in iNKT-deficient mice, gut inflammation was found to be significantly less prominent as compared to WT control mice. Strikingly, the composition of gut microbiota was dramatically altered in iNKT-deficient mice. Moreover, certain types of neutrophiles endowed with anti-inflammatory functions were more frequently recruited in iNKT-deficient mice. Overall, the iNKT cell–microbiota–neutrophil axis was found to play a critical role in regulating gut inflammation [76]. These data suggests that NKT cells are modulated by the host microbiome activity and, conversely, may be involved in the alteration of the gut microbiome composition.

### Macrophages

Macrophages is one of the major cells involved in innate immunity, with M1 phenotypes playing an inflammatory role and M2 phenotypes participating in anti-inflammatory functions [77, 78]. Specifically in the gut, tissue-resident macrophages – traditionally of M2 nature – are less responsive to lipopolysaccharide (LPS) stimulation and produce lower levels of pro-inflammatory cytokines including interleukin (IL)-1β, IL-6 and TNF-α when compared to circulating or blood monocytes [79, 80]. Tumor-associated macrophages (TAMs) are M2 polarized macrophages that produce chemokines and cytokines in the tumor microenvironment to repress cytotoxic T cell activities and induce cancer progression and metastasis [81]. Microbial dysbiosis was found to induce M2 phenotype, promoting the formation of an immunosuppressive milieu and accelerating colonic tumor growth [82]. On the other hand, *Fusobacterium* species were shown to drive colorectal cancer progression by altering the innate immune system and inducing the expression of myeloid-derived suppressor cells and TAMs in the tumor microenvironment, thus suppressing T cell response [83, 84].

### T Lymphocytes

The microbiome critically affects T cell formation and immune responses. For example, segmented filamentous bacteria (SFB), a commensal bacterial microbiota group, is known to be required for the development of intestinal Th17 cells [85]. Furthermore, gut microbiota-derived metabolites, specifically short-chain fatty acids (SCFAs), have been reported to participate in the differentiation of T helper 1 (Th1) or Treg cells [86-88]. Interestingly, microbiota and T cell immunity manifest a critical relationship in the development of autoimmune diseases, including rheumatoid arthritis, type 1 diabetes, obesity, and asthma [89, 90]. Moreover, Bacteriotherapy using commensal bacteria transfer, activate the Treg cells via Myd88 signal pathway, providing the protection effect to food allergy particularly in infants [91]. The gut microbiome is also found to regulate anti-cancer adaptive immune activity, as gut microbiome-depleted mice with pancreatic cancer showed increased levels of CD8^+^ T cells secreting IFN-γ and decreased levels of T cells secreting IL-10 and IL-17 [92].

## General Immune Responses in Cancer

One of the most fundamental roles of the immune system, first suggested half of century ago, is the concept of immunological surveillance of cancer cells in the body [93]. The essential feature was that immune cells would recognize and eliminate tumor cells, similar to the immune protection provided against infectious pathogens. The hypothesis underlying immunological surveillance system of tumor growth was predicted to fail in immunodeficient conditions and to promote increased tumor incidence. However, and strikingly, one study showed that, when tumor cells were inoculated into immunodeficient nude mice there was no significant enhancement of tumor incidence [94]. Nude mice could still generate thymus-independent T cells, though in smaller numbers. Their innate immune system played a compensatory role to support the lack of the adaptive immune system, therefore, providing minimal immunological surveillance against tumor development. To clarify this association, cancer incidence was re-examined in a series of studies using different immunodeficient mice models, including genetic knockouts for RAG2, IFN-γ receptor, or type 1 IFN receptor genes [95]. Interestingly, without prior treatments using carcinogens or crossing with a cancer-prone genetic background, these knockout mice displayed a higher incidence of an invasive adenocarcinoma cancer type throughout their entire lifespan [96]. These results suggest that immunological surveillance is essential to limit tumor incidence, even without prior exposure to carcinogenic environments.

During tumor development and progression, tumor cells avoid the immune system via either repressing the immunological function that may arrest tumor growth or by facilitating the creation of a specific microenvironment that inhibits the tumoricidal functions of immune cells [97, 98]. Several recent studies have demonstrated that antigen-specific T cells transferred into tumor-bearing mice were rapidly turn into an anergic status [99, 100]. These results show that tumor cells provide a permissive microenvironment that renders tumor T cell tolerance to escape immunological surveillance. The mechanisms by which Treg cells control the immune system have been shown to rely on the production of IL-10 and transforming growth factor (TGF)-β [101]. In fact, several animal studies revealed that enhanced Treg expansion potentially causes an impaired anti-tumor immunity [102, 103]. The key mechanisms of immune invasion in cancer are the signal transducer and activator of transcription 3 (STAT3) pathway and myeloid suppressor cells (MSCs) [104-106]. STAT3 signaling plays a critical role in MSC development within the tumor microenvironment. In turn, MSCs produce high levels of reactive oxygen species (ROS) and/or reactive nitrogen species (RNS) that inhibit T cell responses in the tumor area [107-110].

## Microbiome-Immune Axis Effects on Cancer

### Colorectal Cancer (CRC)

Fairly robust evidence supports the idea that gut microbial dysbiosis contributes to carcinogenesis in CRC [111-113]. Indeed, tumor microbiota found in the CRC region was shown to be distinctly different from the adjacent healthy mucosa [114-116]. Preclinical data support this notion, stool samples, which include tumor microbiome, from CRC patients, were transferred into conventional mice influence the induction of polyp formation and enhance the expression of procarcinogenic signals and alter the local immune niche [117]. Apart from this dysbiosis-inducing carcinogenesis, some bacterial strains were found to stimulate inflammation that can cause carcinogenesis through the secretion of proinflammatory toxins. For example, enterotoxigenic *Bacteroides fragilis* are known to produce such toxins [118-120]. Besides the production of proinflammatory toxins, there are several ways for such bacterial species to induce carcinogenesis: they may increase the production of ROS [121], modulate signaling pathways important for tumor development in human and mouse tumor models [83], or act to prevent antitumor immune functions [122]. Some microbiota species may also produce metabolites, such as colibactin, produced by *E. coli* [123, 124], and cytolethal distending toxin, produced by *Campylobacter jejuni* [125], that directly produce genotoxic effects. Components from *Fusobacterium nucleatum*, including FadA adhesion (FadAc) complex, can also activate the β-catenin-Wnt pathway in human colon cancer to induce oncogenic changes [126].

### Liver Cancer

The microbiome located in the gut has also been shown to engage in other malignancies, such as the hepatocellular carcinoma (HCC) [127]. The liver is constantly exposed to microbial communities located in the intestine through the portal venous system. Their metabolites and byproducts may trigger inflammation and hepatotoxicity, or directly induce carcinogenesis. For instance, the modification of primary bile acids, mainly produced by the liver, into secondary bile acids, such as deoxycholic acid (DCA), by microbiota can lead to increased DNA damage, hepatotoxicity, and carcinogenesis [128]. Furthermore, in mouse models, the accumulation of primary bile acids and secondary bile acids changes the concentration of NKT cells in the liver, which was found to repress primary tumor growth and metastasis [75]. In addition, infectious hepatitis, obesity, non-alcoholic steatohepatitis (NASH) and several other pathologies that may induce inflammation and trigger cirrhosis, possibly leading to the development of HCC, are found to be related to gut microbiota [129].

### Breast Cancer

Gut microbiota was also shown to induce breast carcinogenesis through the manipulation of steroid (estrogen) metabolism, and the regulation of energy metabolism and obesity [130]. In fact, gut microbiota was found to have the ability to modulate the expression profile of circulating estrogens and phytoestrogens, impacting on the emergence of breast cancer [131]. Alongside this microbial influence on metabolism, breast cancer is highly affected by the immunological aspects of the microbiome. Indeed, it was found that fat-rich diets may cause dysbiosis, promoting the growth of *Proteobacteria* phylum including the specieses *E. coli*, *Klebsiella*, *Enterobacter*, *Citrobacter*, and *Fusobacterium nucleatum* and hampering the growth of some phylum *Firmicutes* and *Bacteroidetes* [132]. Notably, probiotic, including abundance of *Lactobacillus reuteri*, contributes the anti-cancer effects, triggering the development of CD4^+^CD25^+^ lymphocytes [133]. Contrast to previous enhancement of anti-tumor effect by microbiota treatment, inoculation of *Fusobacterium nucleatum*, originally known to located oral area, attenuated the accumulation of T lymphocytes including CD4^+^ or CD8^+^ T cells, thus the breast cancer cell growth and metastasis was accelerated [134]. However, robust evidence on the direct link between these bacteria and breast cancer is still needed.

### Lung Cancer

Recent reports indicate the abundance of microbiota in the respiratory tract, which have been shown to contribute to lung tumorigenesis [49, 135]. Metabolites secreted by lung microbiota were found to affect the metabolism, and specifically the oncogenic pathway, of lung cancer cells [136]. Moreover, it has also been described that lung microbiota is involved in shaping the immune microenvironment, which can further support cancer cell growth in the lung [136]. Generally, the immune circuit within lung resident immune cells contributes to the homeostasis of respiratory tract tissues during steady states, playing a critical role as a first immune protector against foreign pathogens [137]. Recently, it has been reported that chronic inflammation in the lung tissue is closely related to cancer development due to the accumulation of inflammatory cells, cytokines, chemokines, angiogenesis, and metastasis [138]. Despite the existence of several studies addressing the causes underlying chronic lung inflammation, which may trigger tumorigenesis, the mechanisms are yet to be elucidated.

Several articles have demonstrated the role of lung microbiota in lung tumor development. Similar to the gut, the accumulation of bacterial load and altered bacterial diversity in the airway cause an increase in the production of pro-inflammatory cytokines, including IL-1β and IL-23. Then, these cytokines induce the expansion of lung-resident γδ T cells which, in turn, promote the neutrophil-induced inflammation within the tumor microenvironment. Finally, cytokines derived from infiltrated neutrophils, such as IL-22 and amphiregulin, contribute to the proliferation of lung cancer cells [139, 140]. The specific composition of lung microbiota might be a key player in controlling lung local inflammation in specific microenvironments such as the one seen in lung cancers. Specifically, it has been reported that alterations in the lung bacterial balance from *Firmicutes* to *Proteobacteria* is closely associated to increased anti-tumor immunity after antibiotic treatment [140]. Additionally, the altered bacterial composition caused by specific bacteria taxa, such as *Prevotella* and *Veillonella*, originally located in the oral site, was closely associated to lung inflammation through the increase in the expression of Th17 cells and inflammatory cytokines [141]. Altogether, alterations or imbalances in the bacterial composition in the lung may be considered a potential triggering factor of local chronic inflammation.

## Microbiome and Therapeutic Approach on Cancer

**Fig. 2:**
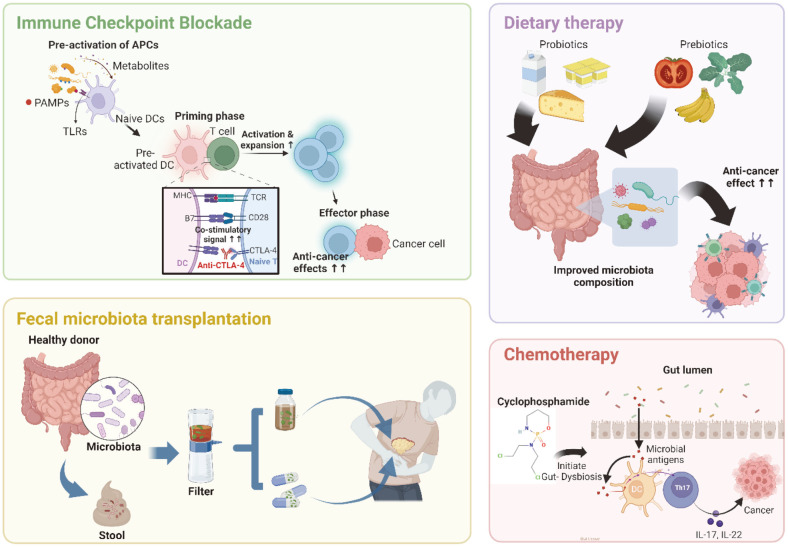
The positive effects of microbiome to the anti-cancer therapeutic methods. Gut microbiota have been well known to contribute to improve the several cancer therapies. At the steady state, certain gut microbiota provides cytokines and PAMPs to activated DCs. It helps to improve the efficacy of immune checkpoint blockade therapy which increase the anticancer function of CD8^+^ T cells. Chemotherapy is one of the old methods to eliminate cancers from patients. Cyclophosphamide has been reported that induce the permeabilization of gut bacteria into lamina propria, thus the infiltrated bacteria activate local immune responses to remove cancers. The direct transfer of microbiota from healthy people to cancer patient via FMT have been proved to improve the condition of cancer patients potentially. Last, the probiotics with live commensal bacteria and the prebiotic which assist the expansion of gut microbiota can help the anti-cancer effects through activate the local immune systems.

Gut microbiota not only participates in carcinogenesis but may also contribute to cancer therapy. The microbiome is found to influence the response and toxicity of various therapeutic approaches, and the dynamics of chemotherapy and immune checkpoint blockade strategies (Fig. 2).

### Immune Checkpoint Blockade Immunotherapy

The idea, immune checkpoint blockade immunotherapy, is based on the amplification of cytotoxic T cells via blocking the checkpoint surface proteins such as CTLA-4 or PD-1 to kill the target cancer cells efficiently [142]. Several studies have proven that gut microbiota contribute to the modulation of tumor responses to immune checkpoint blockade immunotherapies in some cancers [143-146]. Results from clinical models demonstrate that some specific microbial signatures in cancer patients favor systemic immunity and intratumoral immune infiltrates, thus increasing the effects of checkpoint blockade immunotherapy. These results are also supported by preclinical studies employing fecal microbiota transplants (FMT) in germ-free mouse models [144]. Preclinical and clinical studies suggest that among the different features of microbiota-exerting influences on antitumoral immunity, the interaction between microbial components or products (*e.g.*, pathogen-associated molecular patterns (PAMPs)) and the innate immunity including APCs mainly promotes the adaptive immune response [71, 147]. Therefore, those enhanced immune responses, resulting in increasing the anti-tumoral function of cytotoxic T cells which infiltrated in the tumor [145, 146]. These results suggest that further studies need to be focused on identifying the specific bacterial species that may favor antitumoral responses.

### Chemotherapy

Several preclinical models suggest that microbiota influence responses to chemotherapies. In the case of cyclophosphamide, the composition of microbiota is altered and intestinal permeability enhanced, which allows the translocation of specific bacteria into secondary lymphoid organs that stimulate the process of Th17 maturation within the lamina propria and effector lymph nodes [148]. Similarly, responses to local CpG oligonucleotide immunotherapy and oxaliplatin chemotherapy, however, were found to be dependent on microbial-related aspects, namely in the expression of inflammation-promoting genes and the production of ROS by myeloid cells in the tumor microenvironment [149].

### Gut Microbiota and Treatment Toxicity

Gut microbiota not only contributes to therapeutic responses, but it has also been demonstrated to play a role in regulating cancer treatment toxicity. In the case of allogeneic stem cell transplantation, performed for various hematologic malignancies, distinct compositions of gut microbiota yield differential risks of developing graft-versus-host-disease (GVHD) [150-152]. While sites where acute GVHD most commonly occurs are highly occupied by bacterial flora, the development of GVHD has been shown to be related to TLR signaling, implying the critical role of microbiota effects [153, 154]. Gut microbiota also influences treatment toxicity in several other anticancer therapies. Some gut microbial taxa, including bacteria belonging to the *Bacteroidetes* phylum, are found to be protective against immunotherapy toxicity and are more frequently present in patients that are resistant to ipilimumab-induced colitis [155]. Bacteria belonging to the *Bifidobacterium* genus are also involved in fighting some pathological features in an immunotherapy-induced colitis mouse model [156]. Similarly, bacteria belonging to the *Firmicutes* phylum can play a role on immunotherapy and immunotherapy-induced colitis, with several bacterial taxa being related with favorable responses and treatment toxicity. Preclinical models demonstrate the dual role of gut microbiota in response to oxaliplatin, a platinum-based chemotherapy drug, contributing to both tumor cytotoxicity and mechanical hyperalgesia by increasing the levels of ROS and proinflammatory cytokines in the dorsal root ganglion [157]. Besides, radiation was found to modify gut microbial composition in preclinical models, characterized by a reduced abundance of *Firmicutes* and an increased abundance of *Proteobacteria*, with this alteration possibly enhancing the susceptibility to radiation-induced colitis [158].

### Fecal Microbiota Transplantation (FMT)

FMT was a method developed to restore gut microbiota diversity of patients that display different pathologies through the transplantation of fecal matter from healthy people into the intestinal tract of recipients [159, 160]. Historically, the healthy donor’s fecal content was introduced to rescue food poisoning or diarrhea about 1,700 years ago [161]. Following the application of FMT, patients with gastric cancer were found to exhibit a different bacterial diversity and a relative abundance compared to healthy controls, showing a potential prediction of the dysbiotic microbial community [162]. Interestingly, *Helicobacter pylori* has been demonstrated as a major player in FMT strategies, with eradication treatments greatly counteracting the development of gastric adenocarcinoma whereas patients receiving *H. pylori* treatment showing lower rates of metachronous gastric cancer [163, 164].

FMT has been shown to exert great benefits in liver diseases. Indeed, in mouse models, increasing gut microbiota diversity through FMT, alleviates high-fat diet-induced liver damage [165]. Similarly, FMT using fecal matter from alcoholic liver disease resistant donors to recipient mice was found to prevent the progress of alcohol-related liver damage [166]. Furthermore, one pilot study with human patients reported that FMT showed efficacy in improving gut dysbiosis and clinical outcomes in patients with the severe alcoholic hepatitis [167].

### Dietary Therapy

Various microbial consortia are intimately related to human digestion and nutrient uptake. Among them, the gut microbiota is known to be the most important player because it regulates nutritional availability and, in turn, its composition is modulated by diet. Several studies have demonstrated the role of dietary modulation in shaping gut microbial composition. For example, the elimination of animal fat from diet was found to be related to a decrease in bacteria from the *Bacteroidales* order [40] whereas a high-fiber diet was related to short chain fatty acid (SCFA)-producing bacteria [168, 169]. Such modulations likely reflect changes in both mice immunity and human metabolism. Considering this, dietary modulation is thought to play an important role in cancer therapies [170, 171].

Prebiotics and probiotics can also be used to modify and regulate gut microbiota [172]. Prebiotics are specific chemicals that selectively promote the growth of targeted groups of bacteria. Animal studies show that mice with prebiotic-rich diets display enhanced effects of the chemotherapeutics and of radiotherapy [173]. *Lactobacillus acidophilus* as probiotics was first reported that it provides the anti-cancer function to reduce colon cancer compared to healthy control [174]. The live probiotic strains including *Enterococcus faecium* RM11 and *Lactobacillus fermentum* RM28 from milk have been demonstrated that it causes the anti-proliferation of colon cancer cells in vitro condition [175]. At the phase 1 clinical trial, “a bifidogenic-live bacterial probiotics” showed that the improvement of clinical outcome in the renal cell carcinoma patients when co-treatment with checkpoint inhibitors such as nivolumab and ipilimumab [176].

## Conclusions and Outlook

It has been increasingly clear that commensal microbiota greatly contributes to the regulation of human health, mostly by affecting immunity and the immunological landscape. In fact, disruptions in microbial communities, particularly alterations in their diversity, may trigger the development of several pathologies, including autoimmune diseases, allergy, and cancers. Robust evidence suggests that microbiota dysregulation at the community level, but also at the individual level, may underly the genesis of several cancer types, while providing promising avenues for cancer treatment. While microbiota per se can impact cancer outcome, numerous ongoing studies are now addressing the impact of external forces such as diet, antigen exposure, medications, and stress, which may greatly affect the microbiome-immune-cancer axis.

Here we reviewed evidence pointing to the strong effects of a microbiome and tissue immunity relationship in the development and treatment of different types of cancers. However, despite of its impact, we still need to consider some cautious factors. First, in human organs, the microbiome composition among different individuals is too varied, which difficulties the application of a one-size-fits-all therapeutic method. Second, it is still not clear which epigenetic factors play a role in modifying the composition and abundance of microbiota. Current literature extensively suggests the positive effects of FMT strategies in curing specific diseases, in both animal models and human studies, but there are still concerns about how they affect the microbiota landscape of the offspring and about the long-term impact of such strategies. Therefore, further studies focused on understanding the functions and features of microbiome and tissue immune mechanisms are required to overcome such questions. Finally, we believe that advancements on the knowledge about the microbiome-immune axis will provide key insights to improve current cancer therapies in the near future.

## References

[1] Viale PH (2020). The American Cancer Society's Facts & Figures: 2020 Edition. J. Adv. Pract. Oncol..

[2] Siegel RL, Miller KD, Jemal A (2019). Cancer statistics, 2019. CA Cancer J. Clin..

[3] Islami F, Goding Sauer A, Miller KD, Siegel RL, Fedewa SA, Jacobs EJ (2018). Proportion and number of cancer cases and deaths attributable to potentially modifiable risk factors in the United States. CA Cancer J. Clin..

[4] Bouvard V, Baan R, Straif K, Grosse Y, Secretan B, El Ghissassi F (2009). A review of human carcinogens--Part B: biological agents. Lancet Oncol..

[5] Vinay DS, Ryan EP, Pawelec G, Talib WH, Stagg J, Elkord E (2015). Immune evasion in cancer: mechanistic basis and therapeutic strategies. Semin. Cancer Biol..

[6] Togashi Y, Shitara K, Nishikawa H (2019). Regulatory T cells in cancer immunosuppression - implications for anticancer therapy. Nat. Rev. Clin. Oncol..

[7] Tanaka A, Sakaguchi S (2017). Regulatory T cells in cancer immunotherapy. Cell Res..

[8] Itahashi K, Irie T, Nishikawa H (2022). Regulatory T-cell development in the tumor microenvironment. Eur. J. Immunol..

[9] Chen BJ, Zhao JW, Zhang DH, Zheng AH, Wu GQ (2022). Immunotherapy of cancer by targeting regulatory T cells. Int. Immunopharmacol..

[10] Fan Y, Pedersen O (2021). Gut microbiota in human metabolic health and disease. Nat. Rev. Microbiol..

[11] Sorbara MT, Pamer EG (2022). Microbiome-based therapeutics. Nat. Rev. Microbiol..

[12] Gilbert JA, Blaser MJ, Caporaso JG, Jansson JK, Lynch SV, Knight R (2018). Current understanding of the human microbiome. Nat. Med..

[13] Hooper LV, Littman DR, Macpherson AJ (2012). Interactions between the microbiota and the immune system. Science.

[14] Zheng D, Liwinski T, Elinav E (2020). Interaction between microbiota and immunity in health and disease. Cell Res..

[15] Lazar V, Ditu LM, Pircalabioru GG, Gheorghe I, Curutiu C, Holban AM (2018). Aspects of gut microbiota and immune system interactions in infectious diseases, immunopathology, and cancer. Front. Immunol..

[16] Marchesi JR, Ravel J (2015). The vocabulary of microbiome research: a proposal. Microbiome.

[17] Berg G, Rybakova D, Fischer D, Cernava T, Verges MC, Charles T (2020). Microbiome definition re-visited: old concepts and new challenges. Microbiome.

[18] Cho I, Blaser MJ (2012). The human microbiome: at the interface of health and disease. Nat. Rev. Genet..

[19] Blum HE (2017). The human microbiome. Adv. Med. Sci..

[20] Malla MA, Dubey A, Kumar A, Yadav S, Hashem A, Abd Allah EF (2018). Exploring the human microbiome: the potential future role of next-generation sequencing in disease diagnosis and treatment. Front. Immunol..

[21] Wensel CR, Pluznick JL, Salzberg SL, Sears CL (2022). Next-generation sequencing: insights to advance clinical investigations of the microbiome. J. Clin. Invest..

[22] Finotello F, Mastrorilli E, Di Camillo B (2018). Measuring the diversity of the human microbiota with targeted next-generation sequencing. Brief Bioinform..

[23] Browne HP, Forster SC, Anonye BO, Kumar N, Neville BA, Stares MD (2016). Culturing of 'unculturablé human microbiota reveals novel taxa and extensive sporulation. Nature.

[24] Backhed F, Ley RE, Sonnenburg JL, Peterson DA, Gordon JI (2005). Host-bacterial mutualism in the human intestine. Science.

[25] Gill SR, Pop M, Deboy RT, Eckburg PB, Turnbaugh PJ, Samuel BS (2006). Metagenomic analysis of the human distal gut microbiome. Science.

[26] Sender R, Fuchs S, Milo R (2016). Revised estimates for the number of human and bacteria cells in the body. PLoS Biol..

[27] Morais LH, Schreiber HLt, Mazmanian SK (2021). The gut microbiota-brain axis in behaviour and brain disorders. Nat. Rev. Microbiol..

[28] Tripathi A, Debelius J, Brenner DA, Karin M, Loomba R, Schnabl B (2018). The gut-liver axis and the intersection with the microbiome. Nat. Rev. Gastroenterol. Hepatol..

[29] Budden KF, Gellatly SL, Wood DL, Cooper MA, Morrison M, Hugenholtz P (2017). Emerging pathogenic links between microbiota and the gut-lung axis. Nat. Rev. Microbiol..

[30] O'Mahony SM, Clarke G, Borre YE, Dinan TG, Cryan JF (2015). Serotonin, tryptophan metabolism and the brain-gut-microbiome axis. Behav. Brain Res..

[31] Yu G, Gail MH, Consonni D, Carugno M, Humphrys M, Pesatori AC (2016). Characterizing human lung tissue microbiota and its relationship to epidemiological and clinical features. Genome Biol..

[32] Rizzo AE, Gordon JC, Berard AR, Burgener AD, Avril S (2021). The female reproductive tract microbiome-implications for gynecologic cancers and personalized medicine. J. Pers. Med..

[33] Ravel J, Gajer P, Abdo Z, Schneider GM, Koenig SS, McCulle SL (2011). Vaginal microbiome of reproductive-age women. Proc. Natl. Acad. Sci. USA 108 Suppl.

[34] Eslami S, Hadjati J, Motevaseli E, Mirzaei R, Farashi Bonab S, Ansaripour B (2016). *Lactobacillus crispatus* strain SJ-3C-US induces human dendritic cells (DCs) maturation and confers an anti-inflammatory phenotype to DCs. APMIS.

[35] Jain T, Sharma P, Are AC, Vickers SM, Dudeja V (2021). New insights into the cancer-microbiome-immune axis: decrypting a decade of discoveries. Front. Immunol..

[36] Sepich-Poore GD, Zitvogel L, Straussman R, Hasty J, Wargo JA, Knight R (2021). The microbiome and human cancer. Science.

[37] Helmink BA, Khan MAW, Hermann A, Gopalakrishnan V, Wargo JA (2019). The microbiome, cancer, and cancer therapy. Nat. Med..

[38] Bray F, Ferlay J, Soerjomataram I, Siegel RL, Torre LA, Jemal A (2018). Global cancer statistics 2018: GLOBOCAN estimates of incidence and mortality worldwide for 36 cancers in 185 countries. CA Cancer J. Clin..

[39] Qin J, Li R, Raes J, Arumugam M, Burgdorf KS, Manichanh C (2010). A human gut microbial gene catalogue established by metagenomic sequencing. Nature.

[40] Turnbaugh PJ, Ley RE, Mahowald MA, Magrini V, Mardis ER, Gordon JI (2006). An obesity-associated gut microbiome with increased capacity for energy harvest. Nature.

[41] Chung H, Pamp SJ, Hill JA, Surana NK, Edelman SM, Troy EB (2012). Gut immune maturation depends on colonization with a host-specific microbiota. Cell.

[42] Maruvada P, Leone V, Kaplan LM, Chang EB (2017). The human microbiome and obesity: moving beyond associations. Cell Host Microbe..

[43] Reddy BS, Weisburger JH, Narisawa T, Wynder EL (1974). Colon carcinogenesis in germ-free rats with 1,2-dimethylhydrazine and N-methyl-n'-nitro-N-nitrosoguanidine. Cancer Res..

[44] Reddy BS, Narisawa T, Wright P, Vukusich D, Weisburger JH, Wynder EL (1975). Colon carcinogenesis with azoxymethane and dimethylhydrazine in germ-free rats. Cancer Res..

[45] Onoue M, Kado S, Sakaitani Y, Uchida K, Morotomi M (1997). Specific species of intestinal bacteria influence the induction of aberrant crypt foci by 1,2-dimethylhydrazine in rats. Cancer Lett..

[46] Sears CL, Geis AL, Housseau F (2014). *Bacteroides fragilis* subverts mucosal biology: from symbiont to colon carcinogenesis. J. Clin. Invest..

[47] Swidsinski A, Khilkin M, Kerjaschki D, Schreiber S, Ortner M, Weber J (1998). Association between intraepithelial *Escherichia coli* and colorectal cancer. Gastroenterology.

[48] Ramirez-Labrada AG, Isla D, Artal A, Arias M, Rezusta A, Pardo J (2020). The influence of lung microbiota on lung carcinogenesis, immunity, and immunotherapy. Trends Cancer.

[49] Liu NN, Ma Q, Ge Y, Yi CX, Wei LQ, Tan JC (2020). Microbiome dysbiosis in lung cancer: from composition to therapy. NPJ Precis Oncol..

[50] Liu HX, Tao LL, Zhang J, Zhu YG, Zheng Y, Liu D (2018). Difference of lower airway microbiome in bilateral protected specimen brush between lung cancer patients with unilateral lobar masses and control subjects. Int. J. Cancer.

[51] Liu Y, O'Brien JL, Ajami NJ, Scheurer ME, Amirian ES, Armstrong G (2018). Lung tissue microbial profile in lung cancer is distinct from emphysema. Am. J. Cancer Res..

[52] Cameron SJS, Lewis KE, Huws SA, Hegarty MJ, Lewis PD, Pachebat JA (2017). A pilot study using metagenomic sequencing of the sputum microbiome suggests potential bacterial biomarkers for lung cancer. PLoS One.

[53] Yan X, Yang M, Liu J, Gao R, Hu J, Li J (2015). Discovery and validation of potential bacterial biomarkers for lung cancer. Am. J. Cancer Res..

[54] Lee SH, Sung JY, Yong D, Chun J, Kim SY, Song JH (2016). Characterization of microbiome in bronchoalveolar lavage fluid of patients with lung cancer comparing with benign mass like lesions. Lung Cancer.

[55] Sims TT, El Alam MB, Karpinets TV, Dorta-Estremera S, Hegde VL, Nookala S (2021). Gut microbiome diversity is an independent predictor of survival in cervical cancer patients receiving chemoradiation. Commun. Biol..

[56] Riquelme E, Zhang Y, Zhang L, Montiel M, Zoltan M, Dong W (2019). Tumor microbiome diversity and composition influence pancreatic cancer outcomes. Cell.

[57] Chao XP, Sun TT, Wang S, Fan QB, Shi HH, Zhu L (2019). Correlation between the diversity of vaginal microbiota and the risk of high-risk human papillomavirus infection. Int. J. Gynecol. Cancer.

[58] Keller MJ, Huber A, Espinoza L, Serrano MG, Parikh HI, Buck GA (2019). Impact of Herpes simplex virus type 2 and human immunodeficiency virus dual infection on female genital tract mucosal immunity and the vaginal microbiome. J. Infect. Dis..

[59] Liu CM, Packman ZR, Abraham AG, Serwadda DM, Nalugoda F, Aziz M (2019). The effect of antiretroviral therapy initiation on the vaginal microbiome in HIV-infected women. Open Forum Infect. Dis..

[60] Jiang N, Xie F, Chen L, Chen F, Sui L (2020). The effect of TLR4 on the growth and local inflammatory microenvironment of HPVrelated cervical cancer in vivo. Infect. Agent Cancer.

[61] Chao X, Sun T, Wang S, Tan X, Fan Q, Shi H (2020). Research of the potential biomarkers in vaginal microbiome for persistent high-risk human papillomavirus infection. Ann. Transl. Med..

[62] Blanco R, Carrillo-Beltran D, Munoz JP, Corvalan AH, Calaf GM, Aguayo F (2021). Human Papillomavirus in breast carcinogenesis: A passenger, a cofactor, or a causal agent?. Biology (Basel).

[63] Sher G, Salman NA, Kulinski M, Fadel RA, Gupta VK, Anand A (2020). Prevalence and type distribution of high-risk human papillomavirus (HPV) in breast cancer: A qatar based study. Cancers (Basel).

[64] Di Lonardo A, Venuti A, Marcante ML (1992). Human papillomavirus in breast cancer. Breast Cancer Res. Treat..

[65] Kan CY, Iacopetta BJ, Lawson JS, Whitaker NJ (2005). Identification of human papillomavirus DNA gene sequences in human breast cancer. Br. J. Cancer.

[66] Heng B, Glenn WK, Ye Y, Tran B, Delprado W, Lutze-Mann L (2009). Human papilloma virus is associated with breast cancer. Br. J. Cancer.

[67] Nejman D, Livyatan I, Fuks G, Gavert N, Zwang Y, Geller LT (2020). The human tumor microbiome is composed of tumor typespecific intracellular bacteria. Science.

[68] Thompson KJ, Ingle JN, Tang X, Chia N, Jeraldo PR, Walther-Antonio MR (2017). A comprehensive analysis of breast cancer microbiota and host gene expression. PLoS One.

[69] Olszak T, An D, Zeissig S, Vera MP, Richter J, Franke A (2012). Microbial exposure during early life has persistent effects on natural killer T cell function. Science.

[70] Zhang D, Chen G, Manwani D, Mortha A, Xu C, Faith JJ (2015). Neutrophil ageing is regulated by the microbiome. Nature.

[71] Paulos CM, Wrzesinski C, Kaiser A, Hinrichs CS, Chieppa M, Cassard L (2007). Microbial translocation augments the function of adoptively transferred self/tumor-specific CD8+ T cells via TLR4 signaling. J. Clin. Invest..

[72] Uribe-Herranz M, Rafail S, Beghi S, Gil-de-Gomez L, Verginadis I, Bittinger K (2020). Gut microbiota modulate dendritic cell antigen presentation and radiotherapy-induced antitumor immune response. J. Clin. Invest..

[73] Schaupp L, Muth S, Rogell L, Kofoed-Branzk M, Melchior F, Lienenklaus S (2020). Microbiota-induced type I interferons instruct a poised basal state of dendritic cells. Cell.

[74] Nair S, Dhodapkar MV (2017). Natural killer T cells in cancer immunotherapy. Front. Immunol..

[75] Ma C, Han M, Heinrich B, Fu Q, Zhang Q, Sandhu M (2018). Gut microbiome-mediated bile acid metabolism regulates liver cancer via NKT cells. Science.

[76] Shen S, Prame Kumar K, Stanley D, Moore RJ, Van TTH, Wen SW (2018). Invariant natural killer T cells shape the gut microbiota and regulate neutrophil recruitment and function during intestinal inflammation. Front. Immunol..

[77] Saqib U, Sarkar S, Suk K, Mohammad O, Baig MS, Savai R (2018). Phytochemicals as modulators of M1-M2 macrophages in inflammation. Oncotarget.

[78] Mills CD (2012). M1 and M2 macrophages: Oracles of health and disease. Crit. Rev. Immunol..

[79] Xue J, Schmidt SV, Sander J, Draffehn A, Krebs W, Quester I (2014). Transcriptome-based network analysis reveals a spectrum model of human macrophage activation. Immunity.

[80] Van den Bossche J, Saraber DL (2018). Metabolic regulation of macrophages in tissues. Cell Immunol..

[81] Pathria P, Louis TL, Varner JA (2019). Targeting tumor-associated macrophages in cancer. Trends Immunol..

[82] Pushalkar S, Hundeyin M, Daley D, Zambirinis CP, Kurz E, Mishra A (2018). The pancreatic cancer microbiome promotes oncogenesis by induction of innate and adaptive immune suppression. Cancer Discov..

[83] Kostic AD, Chun E, Robertson L, Glickman JN, Gallini CA, Michaud M (2013). *Fusobacterium nucleatum* potentiates intestinal tumorigenesis and modulates the tumor-immune microenvironment. Cell Host Microbe..

[84] Park HE, Kim JH, Cho NY, Lee HS, Kang GH (2017). Intratumoral *Fusobacterium nucleatum* abundance correlates with macrophage infiltration and CDKN2A methylation in microsatellite-unstable colorectal carcinoma. Virchows Arch..

[85] Yang Y, Torchinsky MB, Gobert M, Xiong H, Xu M, Linehan JL (2014). Focused specificity of intestinal TH17 cells towards commensal bacterial antigens. Nature.

[86] Sun M, Wu W, Chen L, Yang W, Huang X, Ma C (2018). Microbiota-derived short-chain fatty acids promote Th1 cell IL-10 production to maintain intestinal homeostasis. Nat. Commun..

[87] Atarashi K, Tanoue T, Shima T, Imaoka A, Kuwahara T, Momose Y (2011). Induction of colonic regulatory T cells by indigenous Clostridium species. Science.

[88] Geuking MB, Cahenzli J, Lawson MA, Ng DC, Slack E, Hapfelmeier S (2011). Intestinal bacterial colonization induces mutualistic regulatory T cell responses. Immunity.

[89] Lee N, Kim WU (2017). Microbiota in T-cell homeostasis and inflammatory diseases. Exp. Mol. Med..

[90] Salvador R, Zhang A, Horai R, Caspi RR (2020). Microbiota as drivers and as therapeutic targets in ocular and tissue specific autoimmunity. Front. Cell Dev. Biol..

[91] Abdel-Gadir A, Stephen-Victor E, Gerber GK, Noval Rivas M, Wang S, Harb H (2019). Microbiota therapy acts via a regulatory T cell MyD88/RORgammat pathway to suppress food allergy. Nat. Med..

[92] Sethi V, Kurtom S, Tarique M, Lavania S, Malchiodi Z, Hellmund L (2018). Gut Microbiota promotes tumor growth in mice by modulating immune response. Gastroenterology.

[93] Burnet FM (1970). The concept of immunological surveillance. Prog. Exp. Tumor Res..

[94] Rygaard J, Povlsen CO (1976). The nude mouse vs. the hypothesis of immunological surveillance. Transplant. Rev..

[95] Dunn GP, Koebel CM, Schreiber RD (2006). Interferons, immunity and cancer immunoediting. Nat. Rev. Immunol..

[96] Shankaran V, Ikeda H, Bruce AT, White JM, Swanson PE, Old LJ (2001). IFNgamma and lymphocytes prevent primary tumour development and shape tumour immunogenicity. Nature.

[97] Tauriello DVF, Sancho E, Batlle E (2022). Overcoming TGFbeta-mediated immune evasion in cancer. Nat. Rev. Cancer.

[98] Kim SK, Cho SW (2022). The evasion mechanisms of cancer immunity and drug intervention in the tumor microenvironment. Front. Pharmacol..

[99] Bogen B (1996). Peripheral T cell tolerance as a tumor escape mechanism: deletion of CD4^+^ T cells specific for a monoclonal immunoglobulin idiotype secreted by a plasmacytoma. Eur. J. Immunol..

[100] Staveley-O'Carroll K, Sotomayor E, Montgomery J, Borrello I, Hwang L, Fein S (1998). Induction of antigen-specific T cell anergy: An early event in the course of tumor progression. Proc. Natl. Acad. Sci. USA.

[101] Vignali DA, Collison LW, Workman CJ (2008). How regulatory T cells work. Nat. Rev. Immunol..

[102] Sutmuller RP, van Duivenvoorde LM, van Elsas A, Schumacher TN, Wildenberg ME, Allison JP (2001). Synergism of cytotoxic T lymphocyte-associated antigen 4 blockade and depletion of CD25(+) regulatory T cells in antitumor therapy reveals alternative pathways for suppression of autoreactive cytotoxic T lymphocyte responses. J. Exp. Med..

[103] Ercolini AM, Ladle BH, Manning EA, Pfannenstiel LW, Armstrong TD, Machiels JP (2005). Recruitment of latent pools of highavidity CD8(+) T cells to the antitumor immune response. J. Exp. Med..

[104] Yu H, Pardoll D, Jove R (2009). STATs in cancer inflammation and immunity: a leading role for STAT3. Nat. Rev. Cancer.

[105] Kusmartsev S, Gabrilovich DI (2006). Role of immature myeloid cells in mechanisms of immune evasion in cancer. Cancer Immunol. Immunother..

[106] Young MR, Petruzzelli GJ, Kolesiak K, Achille N, Lathers DM, Gabrilovich DI (2001). Human squamous cell carcinomas of the head and neck chemoattract immune suppressive CD34(+) progenitor cells. Hum. Immunol..

[107] Mazzoni A, Bronte V, Visintin A, Spitzer JH, Apolloni E, Serafini P (2002). Myeloid suppressor lines inhibit T cell responses by an NO-dependent mechanism. J. Immunol..

[108] Nagaraj S, Gabrilovich DI (2010). Myeloid-derived suppressor cells in human cancer. Cancer J..

[109] Gabrilovich DI, Ostrand-Rosenberg S, Bronte V (2012). Coordinated regulation of myeloid cells by tumours. Nat. Rev. Immunol..

[110] Schmielau J, Finn OJ (2001). Activated granulocytes and granulocyte-derived hydrogen peroxide are the underlying mechanism of suppression of t-cell function in advanced cancer patients. Cancer Res..

[111] Sears CL, Garrett WS (2014). Microbes, microbiota, and colon cancer. Cell Host Microbe..

[112] Yang Y, Jobin C (2017). Novel insights into microbiome in colitis and colorectal cancer. Curr. Opin. Gastroenterol..

[113] Brennan CA, Garrett WS (2016). Gut microbiota, inflammation, and colorectal cancer. Annu. Rev. Microbiol..

[114] Nakatsu G, Li X, Zhou H, Sheng J, Wong SH, Wu WK (2015). Gut mucosal microbiome across stages of colorectal carcinogenesis. Nat. Commun..

[115] Lu Y, Chen J, Zheng J, Hu G, Wang J, Huang C (2016). Mucosal adherent bacterial dysbiosis in patients with colorectal adenomas. Sci. Rep..

[116] Gao Z, Guo B, Gao R, Zhu Q, Qin H (2015). Microbiota disbiosis is associated with colorectal cancer. Front. Microbiol..

[117] Wong SH, Zhao L, Zhang X, Nakatsu G, Han J, Xu W (2017). Gavage of fecal samples from patients with colorectal cancer promotes intestinal Carcinogenesis in Germ-Free and Conventional Mice. Gastroenterology.

[118] Purcell RV, Pearson J, Aitchison A, Dixon L, Frizelle FA, Keenan JI (2017). Colonization with enterotoxigenic bacteroides fragilis is associated with early-stage colorectal neoplasia. PLoS One.

[119] Wu S, Rhee KJ, Albesiano E, Rabizadeh S, Wu X, Yen HR (2009). A human colonic commensal promotes colon tumorigenesis via activation of T helper type 17 T cell responses. Nat. Med..

[120] Boleij A, Hechenbleikner EM, Goodwin AC, Badani R, Stein EM, Lazarev MG (2015). The *Bacteroides fragilis* toxin gene is prevalent in the colon mucosa of colorectal cancer patients. Clin. Infect. Dis..

[121] Mangerich A, Knutson CG, Parry NM, Muthupalani S, Ye W, Prestwich E (2012). Infection-induced colitis in mice causes dynamic and tissue-specific changes in stress response and DNA damage leading to colon cancer. Proc. Natl. Acad. Sci. USA.

[122] Gur C, Ibrahim Y, Isaacson B, Yamin R, Abed J, Gamliel M (2015). Binding of the Fap2 protein of *Fusobacterium nucleatum* to human inhibitory receptor TIGIT protects tumors from immune cell attack. Immunity.

[123] Dalmasso G, Cougnoux A, Delmas J, Darfeuille-Michaud A, Bonnet R (2014). The bacterial genotoxin colibactin promotes colon tumor growth by modifying the tumor microenvironment. Gut Microbes.

[124] Tomkovich S, Yang Y, Winglee K, Gauthier J, Muhlbauer M, Sun X (2017). Locoregional effects of microbiota in a preclinical model of colon carcinogenesis. Cancer Res..

[125] He Z, Gharaibeh RZ, Newsome RC, Pope JL, Dougherty MW, Tomkovich S (2019). *Campylobacter jejuni* promotes colorectal tumorigenesis through the action of cytolethal distending toxin. Gut..

[126] Rubinstein MR, Wang X, Liu W, Hao Y, Cai G, Han YW (2013). *Fusobacterium nucleatum* promotes colorectal carcinogenesis by modulating E-cadherin/beta-catenin signaling via its FadA adhesin. Cell Host Microbe..

[127] Mima K, Nakagawa S, Sawayama H, Ishimoto T, Imai K, Iwatsuki M (2017). The microbiome and hepatobiliary-pancreatic cancers. Cancer Lett..

[128] Yoshimoto S, Loo TM, Atarashi K, Kanda H, Sato S, Oyadomari S (2013). Obesity-induced gut microbial metabolite promotes liver cancer through senescence secretome. Nature.

[129] Wang R, Tang R, Li B, Ma X, Schnabl B, Tilg H (2021). Gut microbiome, liver immunology, and liver diseases. Cell Mol. Immunol..

[130] Garcia-Castillo V, Sanhueza E, McNerney E, Onate SA, Garcia A (2016). Microbiota dysbiosis: a new piece in the understanding of the carcinogenesis puzzle. J. Med. Microbiol..

[131] Kwa M, Plottel CS, Blaser MJ, Adams S (2016). The intestinal microbiome and estrogen receptor-positive female breast cancer. J. Natl. Cancer Inst..

[132] Shapira I, Sultan K, Lee A, Taioli E (2013). Evolving concepts: how diet and the intestinal microbiome act as modulators of breast malignancy. ISRN Oncol..

[133] Lakritz JR, Poutahidis T, Levkovich T, Varian BJ, Ibrahim YM, Chatzigiagkos A (2014). Beneficial bacteria stimulate host immune cells to counteract dietary and genetic predisposition to mammary cancer in mice. Int. J. Cancer.

[134] Parhi L, Alon-Maimon T, Sol A, Nejman D, Shhadeh A, Fainsod-Levi T (2020). Breast cancer colonization by *Fusobacterium nucleatum* accelerates tumor growth and metastatic progression. Nat. Commun..

[135] Dong Q, Chen ES, Zhao C, Jin C (2021). Host-microbiome interaction in lung cancer. Front. Immunol..

[136] Weinberg F, Dickson RP, Nagrath D, Ramnath N (2020). The lung microbiome: A central mediator of host inflammation and metabolism in lung cancer patients?. Cancers (Basel).

[137] Lloyd CM, Marsland BJ (2017). Lung homeostasis: influence of age, microbes, and the immune system. Immunity.

[138] Palucka AK, Coussens LM (2016). The basis of oncoimmunology. Cell.

[139] Jin C, Lagoudas GK, Zhao C, Bullman S, Bhutkar A, Hu B (2019). Commensal microbiota promote lung cancer development via gammadelta T cells. Cell.

[140] Le Noci V, Guglielmetti S, Arioli S, Camisaschi C, Bianchi F, Sommariva M (2018). Modulation of pulmonary microbiota by antibiotic or probiotic aerosol therapy: a strategy to promote immunosurveillance against lung metastases. Cell Rep..

[141] Segal LN, Clemente JC, Tsay JC, Koralov SB, Keller BC, Wu BG (2016). Enrichment of the lung microbiome with oral taxa is associated with lung inflammation of a Th17 phenotype. Nat. Microbiol..

[142] Postow MA, Callahan MK, Wolchok JD (2015). Immune checkpoint blockade in cancer therapy. J. Clin. Oncol..

[143] Frankel AE, Coughlin LA, Kim J, Froehlich TW, Xie Y, Frenkel EP (2017). Metagenomic shotgun sequencing and unbiased metabolomic profiling identify specific human gut microbiota and metabolites associated with immune checkpoint therapy efficacy in melanoma patients. Neoplasia.

[144] Routy B, Le Chatelier E, Derosa L, Duong CPM, Alou MT, Daillere R (2018). Gut microbiome influences efficacy of PD-1-based immunotherapy against epithelial tumors. Science.

[145] Matson V, Fessler J, Bao R, Chongsuwat T, Zha Y, Alegre ML (2018). The commensal microbiome is associated with anti-PD-1 efficacy in metastatic melanoma patients. Science.

[146] Gopalakrishnan V, Spencer CN, Nezi L, Reuben A, Andrews MC, Karpinets TV (2018). Gut microbiome modulates response to anti-PD-1 immunotherapy in melanoma patients. Science.

[147] Sivan A, Corrales L, Hubert N, Williams JB, Aquino-Michaels K, Earley ZM (2015). Commensal Bifidobacterium promotes antitumor immunity and facilitates anti-PD-L1 efficacy. Science.

[148] Viaud S, Saccheri F, Mignot G, Yamazaki T, Daillere R, Hannani D (2013). The intestinal microbiota modulates the anticancer immune effects of cyclophosphamide. Science.

[149] Iida N, Dzutsev A, Stewart CA, Smith L, Bouladoux N, Weingarten RA (2013). Commensal bacteria control cancer response to therapy by modulating the tumor microenvironment. Science.

[150] van Vliet MJ, Tissing WJ, Dun CA, Meessen NE, Kamps WA, de Bont ES (2009). Chemotherapy treatment in pediatric patients with acute myeloid leukemia receiving antimicrobial prophylaxis leads to a relative increase of colonization with potentially pathogenic bacteria in the gut. Clin. Infect. Dis..

[151] Weber D, Oefner PJ, Hiergeist A, Koestler J, Gessner A, Weber M (2015). Low urinary indoxyl sulfate levels early after transplantation reflect a disrupted microbiome and are associated with poor outcome. Blood.

[152] Jenq RR, Ubeda C, Taur Y, Menezes CC, Khanin R, Dudakov JA (2012). Regulation of intestinal inflammation by microbiota following allogeneic bone marrow transplantation. J. Exp. Med..

[153] Jacobsohn DA, Vogelsang GB (2007). Acute graft versus host disease. Orphanet. J. Rare Dis..

[154] Heimesaat MM, Nogai A, Bereswill S, Plickert R, Fischer A, Loddenkemper C (2010). MyD88/TLR9 mediated immunopathology and gut microbiota dynamics in a novel murine model of intestinal graft-versus-host disease. Gut.

[155] Dubin K, Callahan MK, Ren B, Khanin R, Viale A, Ling L (2016). Intestinal microbiome analyses identify melanoma patients at risk for checkpoint-blockade-induced colitis. Nat. Commun..

[156] Wang F, Yin Q, Chen L, Davis MM (2018). Bifidobacterium can mitigate intestinal immunopathology in the context of CTLA-4 blockade. Proc. Natl. Acad. Sci. USA.

[157] Shen S, Lim G, You Z, Ding W, Huang P, Ran C (2017). Gut microbiota is critical for the induction of chemotherapy-induced pain. Nat. Neurosci..

[158] Sokol H, Adolph TE (2018). The microbiota: an underestimated actor in radiation-induced lesions?. Gut.

[159] van Nood E, Vrieze A, Nieuwdorp M, Fuentes S, Zoetendal EG, de Vos WM (2013). Duodenal infusion of donor feces for recurrent *Clostridium difficile*. N. Engl. J. Med..

[160] Bakken JS, Borody T, Brandt LJ, Brill JV, Demarco DC, Franzos MA (2011). Treating *Clostridium difficile* infection with fecal microbiota transplantation. Clin. Gastroenterol. Hepatol..

[161] Gostin LO, Magnusson RS, Krech R, Patterson DW, Solomon SA, Walton D (2017). Advancing the right to health-the vital role of law. Am. J. Public Health.

[162] Shah MA (2017). Gastric cancer: The gastric microbiota - bacterial diversity and implications. Nat. Rev. Gastroenterol. Hepatol..

[163] Doorakkers E, Lagergren J, Engstrand L, Brusselaers N (2018). *Helicobacter pylori* eradication treatment and the risk of gastric adenocarcinoma in a Western population. Gut.

[164] Choi IJ, Kook MC, Kim YI, Cho SJ, Lee JY, Kim CG (2018). *Helicobacter pylori* Therapy for the prevention of metachronous gastric vancer. N. Engl. J. Med..

[165] Zhou D, Pan Q, Shen F, Cao HX, Ding WJ, Chen YW (2017). Total fecal microbiota transplantation alleviates high-fat dietinduced steatohepatitis in mice via beneficial regulation of gut microbiota. Sci. Rep..

[166] Ferrere G, Wrzosek L, Cailleux F, Turpin W, Puchois V, Spatz M (2017). Fecal microbiota manipulation prevents dysbiosis and alcohol-induced liver injury in mice. J. Hepatol..

[167] Philips CA, Pande A, Shasthry SM, Jamwal KD, Khillan V, Chandel SS (2017). Healthy donor fecal microbiota transplantation in steroid-ineligible severe alcoholic hepatitis: A pilot study. Clin. Gastroenterol. Hepatol..

[168] Zhao L, Zhang F, Ding X, Wu G, Lam YY, Wang X (2018). Gut bacteria selectively promoted by dietary fibers alleviate type 2 diabetes. Science.

[169] Trompette A, Gollwitzer ES, Pattaroni C, Lopez-Mejia IC, Riva E, Pernot J (2018). Dietary fiber confers protection against flu by shaping Ly6c(-) patrolling monocyte hematopoiesis and CD8(+) T cell metabolism. Immunity.

[170] Wu GD, Chen J, Hoffmann C, Bittinger K, Chen YY, Keilbaugh SA (2011). Linking long-term dietary patterns with gut microbial enterotypes. Science.

[171] Cotillard A, Kennedy SP, Kong LC, Prifti E, Pons N, Le Chatelier E (2013). Dietary intervention impact on gut microbial gene richness. Nature.

[172] Sanders ME, Merenstein DJ, Reid G, Gibson GR, Rastall RA (2019). Probiotics and prebiotics in intestinal health and disease: from biology to the clinic. Nat. Rev. Gastroenterol. Hepatol..

[173] Wang H, Geier MS, Howarth GS (2016). Prebiotics: a potential treatment strategy for the chemotherapy-damaged gut?. Crit. Rev. Food Sci. Nutr..

[174] Goldin BR, Gorbach SL (1980). Effect of *Lactobacillus acidophilus* dietary supplements on 1,2-dimethylhydrazine dihydrochlorideinduced intestinal cancer in rats. J. Natl. Cancer Inst..

[175] Thirabunyanon M, Boonprasom P, Niamsup P (2009). Probiotic potential of lactic acid bacteria isolated from fermented dairy milks on antiproliferation of colon cancer cells. Biotechnol. Lett..

[176] Dizman N, Meza L, Bergerot P, Alcantara M, Dorff T, Lyou Y (2022). Nivolumab plus ipilimumab with or without live bacterial supplementation in metastatic renal cell carcinoma: a randomized phase 1 trial. Nat. Med..

